# Association of the Mayo-Adhesive Probability Score With the Total Operative Time of Hand-Assisted Laparoscopic Donor Nephrectomy

**DOI:** 10.7759/cureus.76166

**Published:** 2024-12-21

**Authors:** Waqas Rahim, Liaqat Ullah, Muhammad Ismail Asim, Mubashar Naseer, Muhammad Bilal Anwar, Muhammad Hassan Azad, Adnan Khan, Raza Ashraf

**Affiliations:** 1 Urology, Shifa International Hospital, Islamabad, PAK; 2 Surgery, Shifa International Hospital, Islamabad, PAK; 3 Surgery, Shifa Tameer-E-Millat University Shifa College of Medicine, Islamabad, PAK

**Keywords:** hand-assisted laparoscopic nephrectomy, kidney transplantation, laparoscopic donor nephrectomy, live donor nephrectomy, mayo adhesive probability score

## Abstract

Introduction: Living-donor kidney transplantation (LDKT) is often performed using hand-assisted laparoscopic donor nephrectomy (HALDN). Adherent perinephric fat (APF) can complicate HALDN, increasing operative time. The Mayo Adhesive Probability (MAP) score predicts APF preoperatively. This study investigates the association between MAP score and operative time in patients undergoing HALDN.

Methodology: This cross-sectional study included 133 patients undergoing HALDN at Shifa International Hospital, Islamabad, Pakistan, from December 2021 to July 2023. The primary outcome was total operative time, defined as incision-to-closure duration. The predictor of interest was the MAP score, calculated from preoperative CT scans assessing posterior renal fat thickness and perinephric fat stranding. Data collection included demographic and clinical characteristics of the patients. Data analysis was done using IBM SPSS Statistics software, version 25 (IBM Corp., Armonk, NY), to determine a significant association between the mean operative time across different MAP scores and other parameters, taking a p-value <0.05 as significant.

Results: The mean donor age was 35.1 ± 10.1 years, and females were predominant (85, 63.9%). Most patients had a MAP score of 0 (76.7%) and an American Society of Anesthesiologists (ASA) score of one (81.2%). The mean operative time was 196.86 + 53.81 minutes. The MAP score was not significantly associated with operative time (p = 0.244). Mean operative time did not significantly differ across MAP score groups (p = 0.148). There was a significant association between gender and MAP score, with females having lower scores (p = 0.001). No significant correlations were found between operative time and MAP score, gender, or ASA score (p > 0.05).

Conclusions: The MAP score does not significantly correlate with operative time in HALDN among the studied population. Interestingly, a significant association was noted between lower MAP scores and female gender, adding to the understanding of gender-specific characteristics in laparoscopic donor nephrectomy and highlighting the need for further research to validate the utility of this score in diverse clinical settings and populations. These results underline the need for larger, multicentered studies to validate the utility of the MAP score in predicting operative complexity across diverse clinical settings and populations. Our study contributes to the ongoing efforts to optimize preoperative planning and enhance outcomes in LDKT cases.

## Introduction

Living-donor kidney transplantation (LDKT) is a life-saving treatment for patients with end-stage renal disease. Hand-assisted laparoscopic donor nephrectomy (HALDN) has become the preferred approach for LDKT, with approximately 60% of procedures now performed via HALDN due to its minimally invasive nature and associated benefits for donors, including faster recovery and improved cosmetic outcomes [1]. However, surgical complexity can vary during HALDN, impacting operative time and potentially donor outcomes [2]. 

One factor influencing surgical complexity in HALDN is the presence of adherent perinephric fat (APF) which is a type of inflammatory fat that surrounds the kidney and can increase surgical complexity and extend operative times during partial nephrectomy [3, 4]. Preoperatively predicting the presence and extent of APF is crucial for surgical planning and potentially optimizing operative time. Adherent perinephric fat can be preoperatively predicted using several specific clinical and radiographic factors, including male gender, higher BMI, and the Mayo Adhesive Probability (MAP) score [3, 5]. Patients with APF have longer operative and arterial clamp times than non-APF patients [3]. The presence of APF is associated with an increased operative time, warm ischemia time, and greater estimated blood loss but has no impact on other perioperative outcomes in laparoscopic partial nephrectomy [5].

The MAP score, derived from preoperative imaging (CT scan), has emerged as a reliable tool for predicting APF in both partial and radical nephrectomy and can be used to assess surgical complexity in partial nephrectomy [6]. Notably, the MAP score has emerged as a potential predictor of the procedural challenges encountered during HALDN for LDKT [7]. Studies have demonstrated a positive correlation between MAP scores and the presence and severity of APF. Studies have demonstrated an association between the MAP score and the total operative time, suggesting its relevance in gauging the complexity of laparoscopic donor nephrectomy [8-11]. Therefore, the MAP score is valuable in preoperative assessment and planning for laparoscopic donor nephrectomy, as it can help anticipate the surgical challenges posed by the presence of APF and its impact on operative time [10]. However, the association between MAP score and total operative time in HALDN remains underexplored in the Pakistani population.

This study aimed to investigate the association between donor kidney MAP score and total operative time in patients undergoing HALDN. We hypothesize that a higher MAP score will be associated with increased operative time. Understanding this relationship can have crucial implications for surgical planning, patient counseling, and potentially optimizing resource allocation in the setting of HALDN. This study will contribute valuable insights into the role of MAP score in predicting operative complexity during HALDN, potentially leading to improved surgical efficiency and better donor outcomes.

## Materials and methods

This cross-sectional observational study enrolled 133 patients meeting the inclusion criteria through a nonprobability consecutive sampling technique at the Department of Urology, Shifa International Hospital, Islamabad, Pakistan, conducted from December 2021 to July 2023. Admitted patients provided data after informed consent and data were obtained on a meticulously designed questionnaire. Required ethical approval was obtained from the institutional review board and ethics committee of Shifa International Hospital (approval number: IRB#247-21).

The procedure involved HALDN, executed by fellowship-trained transplant surgeons at a single institution. A standard Gelport device and two additional trocars were employed, with a 5 mm camera port placed at the surgeon's discretion. A 12 mm working port was positioned superior and medial to the iliac spine ipsilateral to the donor kidney. The perinephric fat was dissected, and the hilum was exposed in each case. The ureter was circumferentially dissected and sharply divided distally after control with a clip. An endovascular stapler was utilized to manage the renal artery and vein separately. Gonadal and lumbar veins were clipped and cut when necessary. The kidney was then freed of its attachments and transported to the recipient surgeon.

The primary outcome variable was the total HALDN operative time, defined as the duration from incision to closure. The predictor variable of interest was the MAP score of the donated kidney. The MAP score for the donated kidney was calculated through the assessment of two key features observed in CT scans. These features were posterior renal fat thickness and perinephric stranding. The posterior fat thickness, measured at the level of the renal vein, was scored as <1 cm = 0; 1-1.9 cm = 1; >2 cm = 2. For the perinephric fat stranding, a score of 0 indicated the absence of stranding, two represented mild or equivocal stranding, and three signified definite or severe stranding. The total MAP score was then calculated as the sum of the scores for posterior renal fat and perinephric stranding, resulting in a composite score that ranged from 0 to five. This validated scoring system provided a standardized and qualitative measure [12, 13]. Data collected included patient characteristics (age, sex, race), preoperative laboratory values (body mass index (BMI), estimated glomerular filtration rate (eGFR), hemoglobin (Hgb), platelets, white blood cell count (WBC)), and the sidedness of the donor kidney.

Statistical analysis was performed using IBM SPSS Statistics software, Version 25.0 (IBM Corp., Armonk, NY), calculating means and standard deviations (SDs) for numerical variables such as age, BMI, operative time, preoperative and postoperative hemoglobin, eGFR, platelets, WBC, MAP score, and American Society of Anesthesiologists (ASA) score. Age groups, gender, and donor kidney frequencies and percentages were computed. The Pearson correlation coefficient was employed to assess the correlation between MAP score and operative time, considering a p-value < 0.05 as statistically significant. Results were comprehensively presented through tables and graphs for clarity and accessibility.

## Results

Table 1 shows the descriptive statistics for various clinical parameters of the study participants. The mean age of the donors was 35.12 ± 10.07 years, while the mean operative time was 196.86 ± 53.81 minutes, representing a good variation in the operative time.

**Table 1:** Summary of the clinical characteristics of the study participants (n=133)

Numerical variables		Mean	Standard deviation (SD)
Age		35.12	10.07
Body mass index (BMI)		27.32	4.88
Operative time		196.86	53.81
Preoperative creatinine (Cr)		0.78	0.16
Estimated glomerular filtration rate (eGFR)		98.33	20.88
Preoperative hemoglobin (Hb)		13.40	2.20
White blood cell (WBC)		13348.79	4853.74

Table 2 shows the categorical characteristics of the study participants. The study participants consisted predominantly of females (85, 63.9%), with the left kidney being more commonly donated (129, 97.0%). The majority of the donors had a MAP score of 0 (76.7%), an ASA score of one (81.2%), and were below 50 years of age (93.2%).

**Table 2:** Category-wise characteristics of the study participants

Characteristics		Frequency (n = 133)	Percent
Mayo Adhesive Probability (MAP) score	0	102	76.7
	1	26	19.5
	2	5	3.8
American Society of Anesthesiologists (ASA) score	1	108	81.2
	2	24	18.0
	3	1	0.8
Age categories	< 50 years	124	93.2%
	> 50 years	9	6.8%
Gender	Male	48	36.1%
	Female	85	63.9%
Donor kidney side	Right	4	3.0%
	Left	129	97.0%

Table 3 shows the mean ± standard deviation (SD) for various clinical parameters across different MAP scores. The ANOVA results for each parameter revealed varying degrees of association with MAP scores. Specifically, preoperative creatinine levels were significantly lower for patients with lower MAP scores (F(2, 130) = 6.478, p = 0.002). However, the other parameters, including the mean operative time, did not show statistically significant differences among MAP score groups. The lack of statistical significance suggests that there is no substantial difference in operative times across MAP score categories.

**Table 3:** Comparison of the clinical parameters among Mayo Adhesive Probability (MAP) score categories

Mayo Adhesive Probability (MAP) score of the donated kidney		Age	BMI	Preoperative creatinine	Pre-surgery glomerular filtration rate (GFR)	Preoperative hemoglobin	American Society of Anesthesiologists (ASA) score	Operative time
0 (n=102)	Mean	34.29	27.15	0.75	99.43	13.17	1.186	200.59
	SD	10.01	4.98	0.16	21.95	2.23	0.42	55.16
1 (n=26)	Mean	37.46	27.95	0.87	94.87	14.15	1.23	182.15
	SD	10.10	4.89	0.155	16.91	2.096	0.430	47.02
2 (n=5)	Mean	39.80	27.56	0.86	93.87	14.14	1.200	197.86
	SD	9.68	2.48	0.12	17.39	1.28	0.45	56.12
ANOVA test p-value		0.205	0.753	0.002	0.546	0.098	0.890	0.229

The relationship between gender and the MAP score of the donated kidneys was examined using a cross-tabulation analysis, as shown in Table 4. Female donors had significantly lower MAP scores for the donated kidney. A chi-square test indicated a statistically significant association (χ² = 14.213, df = 2, p = 0.001), suggesting that the distribution of MAP scores differs significantly between male and female participants. Both the likelihood ratio (χ² = 13.786, df = 2, p = 0.001) and the linear-by-linear association (χ² = 11.886, df = 1, p = 0.001) tests supported this finding.

**Table 4:** Cross-tabulation between Mayo Adhesive Probability (MAP) scores and gender

		MAP score of the donated kidney			Total	Chi-square test p-value
		0	1	2		
Gender	Male	28	17	3	48	0.001
	Female	74	9	2	85	

Table 5 summarizes operative time in HALDN across demographic and clinical categories. As shown in the table, no significant difference was noted in the mean operating time between the categories of gender, MAP score, or ASA score. These findings offer insights into procedural time dynamics in different donor and clinical contexts.

**Table 5:** Operative time in hand-assisted laparoscopic donor nephrectomy across demographic and clinical categories

Characteristics		Mean operative time	SD	p-value
Gender	Male (n = 48)	211.50	51.801	(T test) 0.18
	Female (n = 85)	188.59	53.456	
Mayo Adhesive Probability (MAP) categories	MAP = 0 (n = 102)	200.59	55.162	(T test) 0.148
	MAP > 0 (n = 31)	184.58	47.902	
American Society of Anesthesiologists (ASA) score	1 (n = 108)	197.80	55.88	(ANOVA test) 0.788
	2 (n = 24)	191.58	45.11	
	3 (n = 1)	222.00	.	

Table 6 displays the Pearson correlation coefficients examining the relationship between operative time and various clinical parameters. The correlation coefficients range from -0.026 to 0.084, indicating predominantly weak correlations. However, none of the correlations reached statistical significance, as evidenced by p-values ranging from 0.334 to 0.991. These findings suggest that there is no discernible, significant linear association between operative time and the evaluated clinical parameters in our population.

**Table 6:** Correlation between operative time and clinical parameters

Correlation Table		BMI	Mayo Adhesive Probability (MAP) score	American Society of Anesthesiologists (ASA) score	Preoperative creatinine	Preoperative glomerular filtration rate (GFR)	Preoperative hemoglobin	White blood cell count
Operative time	Pearson correlation	-0.065	-0.102	-0.026	0.051	0.045	0.084	0.001
	Sig. (two-tailed)	0.456	0.244	0.768	0.563	0.607	0.334	0.991

## Discussion

The first successes in standard laparoscopic live donor nephrectomy by Ratner and Kavoussi in 1995 and HALDN by Wolf in 1998 have proven the minimal invasiveness of these procedures compared with open donor nephrectomy, including lower morbidity, shorter hospitalization, and rapid convalescence [14]. We investigated the association of the MAP score with the operative time in HALDN, as the literature suggests that the MAP score is significantly associated with significantly longer operative time because the higher MAP score is associated with dense APF [2, 7]. In our study, the mean operative time for patients with a MAP score of 0 was lower (200.59 ± 55.16 minutes) than those with a MAP score > 0 (184.58 ± 47.90 minutes). This finding aligns with the concept that better perinephric fat characteristics (lower MAP scores) may lead to smoother surgeries with less operative time. However, we did not find a statistically significant difference in the operative time between patients with different MAP scores (p = 0.148). This contrasts with published studies that report a positive correlation between higher MAP scores and longer operative times in HALDN [15-18].

Keizu and colleagues studied the difficulty associated with a higher MAP score causing increased operative time to develop a surgical difficulty prediction model [17]. The goal was to be able to preoperatively decide the skill and experience of the surgeon needed to improve outcomes in LDKT. Their scoring system showed a moderately high sensitivity (78.0%) and specificity (64.9%) for predicting surgical difficulty (95% CI = 0.70 to 0.83). Another study also reported that a higher MAP score was associated with the presence of significant APF, thereby predicting a significantly longer duration of the surgery (p = 0.004) and higher estimated blood loss (p = 0.003). Although not statistically significant, their findings did suggest the association of MAP score with postoperative complications and length of hospital stay > three days [18]. In a study by Cockerril et al., donor kidney MAP score > 0 was found to be significantly correlated with longer operative time (an increase of 24.4 minutes, P = .001) in single-variable analysis. This correlation was only significant for males (an increase of 28.9 minutes, P = .013) in multivariable analysis [19]. However, in contrast to the aforementioned studies, we did not find a significant association of MAP score with operative time (p = 0.244).

Possible reasons for the discrepancy include a smaller sample size compared to studies showing a significant association, experience of the surgeon and surgical technique, prior abdominal interventions that may have led to adhesion formation, patient populations, institutional practices, or other unaccounted factors. A larger sample size could provide more power to detect smaller differences in operative times. Surgeon experience can significantly impact operative time. Studies demonstrating a MAP-time association might have involved surgeons with a steeper learning curve [17]. Factors like prior abdominal surgeries or specific conditions affecting adhesions might have been more prevalent in studies showing a MAP-time link, where navigating dense adhesions (high MAP) takes longer [20]. Future research should consider multicenter studies and larger sample sizes to enhance generalizability and comprehensiveness.

We observed a statistically significant association between lower MAP scores and female gender. This aligns with existing literature [17]. Also, preoperative creatinine levels were lower in patients with lower MAP scores, suggesting potential early signs of kidney dysfunction associated with dense adhesions and an association of lower MAP scores with lower preoperative creatinine levels. The majority of donors were females, with a prevalence of left kidney donations and a predominant MAP score of 0. These results contribute valuable insights to the existing literature on gender-specific considerations in laparoscopic donor nephrectomy. A study from Japan reported that the majority of the donors (95%) donated their left kidneys [17]. Studies have consistently reported that the mean MAP score was significantly lower in women, whereas the majority of the donors were also female [7, 17, 20]. The identification of significant associations between gender and MAP scores underscores the importance of individualized surgical planning and highlights the potential impact of patient-specific factors on procedural outcomes.

The strength of this study was the single-surgeon nature of the evaluation to eliminate surgical technique variation as a contributor to outcomes. The limitations of this study were its study design, small sample size, and single center. Large multicenter randomized control trials should be encouraged to get better results so that they can be applied to the overall population.

## Conclusions

Our findings suggest that the MAP score does not have a statistically significant correlation with operative time in HALDN within our population. Interestingly, a significant association was noted between lower MAP scores and female gender, adding to the understanding of gender-specific characteristics in laparoscopic donor nephrectomy. These results underline the need for larger, multicentered studies to validate the utility of the MAP score in predicting operative complexity across diverse clinical settings and populations. Furthermore, incorporating additional factors such as surgical expertise and patient-specific variables could provide a more comprehensive assessment of its predictive value. Our study contributes to the ongoing efforts to optimize preoperative planning and enhance outcomes in LDKT.
